# Optimization of adult mosquito trap settings to monitor populations of *Aedes* and *Culex* mosquitoes, vectors of arboviruses in La Reunion

**DOI:** 10.1038/s41598-022-24191-9

**Published:** 2022-11-15

**Authors:** Iris Claudel, Ronan Brouazin, Renaud Lancelot, Louis-Clément Gouagna, Marlène Dupraz, Thierry Baldet, Jérémy Bouyer

**Affiliations:** 1 UMR Mivegec (Maladies Infectieuses et Vecteurs: Écologie, Génétique, Évolution et Contrôle), IRD-CNRS-Univ. Montpellier, 97410 Saint-Pierre, La Réunion France; 2 UMR Astre (Animals, Health, Territories, Risks, Ecosystems), Cirad, Inrae, Univ. Montpellier, 34398 Montpellier, France; 3 Cirad, UMR Astre, 97491 Sainte Clotilde, La Réunion France; 4 UMR Mivegec, 34394 Montpellier, France; 5 Insect Pest Control Laboratory, Joint FAO/IAEA Programme of Nuclear Techniques in Food and Agriculture, IAEA Vienna, Wagramer Strasse 5, 1400 Vienna, Austria

**Keywords:** Entomology, Ecological epidemiology

## Abstract

Competent arbovirus vectors are found in the culicid mosquito fauna of south-west Indian Ocean (SWIO) islands. In La Reunion, *Aedes albopictus* and *Aedes aegypti* mosquitoes are known vectors of dengue and chikungunya viruses. *Culex quinquefasciatus* is a potential vector of Rift Valley fever and West Nile viruses. To prepare a vector-control field trial against *Ae. aegypti*, this study aimed at identifying the best trapping strategy to catch adult *Ae. aegypti*, using BG-Sentinel traps (Biogents, Germany). It was implemented in two sites in southern La Reunion. Catches of *Ae. albopictus* and *Cx. quinquefasciatus* mosquitoes were also recorded. A Latin square design was used to estimate the detection probability and the apparent daily density—according to the BG-Sentinel trapping strategy: none, carbon dioxide (CO_2_), a commercial attractant—BG-Lure (Biogents, Germany), or both. The use of CO_2_ alone was associated with a higher detection probability for *Ae. aegypti* and *Cx. quinquefasciatus* mosquitoes, as well as a large increase in their apparent density. Traps with BG-Lure—alone or in combination with CO_2_, did not improve the detection probability of *Ae. aegypti* and *Cx. quinquefasciatus* mosquitoes. The same result was found for male *Ae. albopictus*. For females, baiting BG-Sentinel traps with CO_2_ or BG-Lure had no significant effect. The same apparent densities were found for *Ae. aegypti* and *Ae. albopictus* mosquitoes in both study sites—where *Ae. aegypti* mosquitoes were found at very low densities during previous surveys.

## Introduction

Factors favoring the expansion—and increase, of mosquito-borne arboviruses of public health importance are all present in the south-west Indian Ocean (SWIO) islands: human population growth, urbanization, limited natural space (except in Madagascar), high density of vector populations, lack of means and/or effectiveness of vector control methods, increase in maritime and air traffic with strong links between Africa and south-east Asia^1^.

With 12 mosquito species, the culicid fauna of La Reunion is the poorest of the SWIO islands^2^. Populations of *Aedes albopictus* and *Culex quinquefasciatus* are predominant. *Aedes albopictus* was first recorded on the island in early twentieth century. This species is omnipresent, from the coast to 1200 m altitude^3^, in close contact with humans. *Aedes aegypti*, another major arbovirus vector, is also present on La Reunion. It is found as isolated mosquito populations located in *ravines* (narrow valleys) in the west and south of the island. In these ravines, *Ae. aegypti* and *Ae. albopictus* mosquitoes are competing. *Culex quinquefasciatus* is found all along the coastline—up to 2000 m altitude in the summer, particularly in anthropized areas. Its larvae are mainly observed in polluted water. This mosquito was the main vector responsible for Bancroft’s filariasis which was widespread in La Réunion Island in the nineteenth century.

In recent years, dengue epidemics have reached proportions never seen before in this island. Since 2017, La Reunion has been experiencing a dengue epidemic, with increasing intensity from 2018 to 2021. Chikungunya emerged in the Comoros in July 2004, spreading from outbreaks on the East African coast, to all the SWIO islands. In La Reunion, the chikungunya epidemic was spectacular, with 3000 cases recorded during a first wave (2005) and more than 250,000 cases during a second wave (2006), resulting in more than 200 deaths. For these two arboviroses, the main vector in La Reunion is *Ae. albopictus*. However, *Ae. aegypti* can also transmit these viruses even if its current vector role is not considered due to its assumed rarity and isolation. The Zika virus circulated widely in the Pacific between 2007 and 2013 and then in the Caribbean and Latin America in 2015–2016. It might be introduced into the Indian Ocean by viremic travelers and transmitted locally by these two *Aedes* species which are competent for African strains of the Zika virus^4^. However, *Ae. albopictus* is not competent for the Asian strain that invaded the World^5^. Rift Valley fever (RVF) is a zoonotic arbovirosis infecting humans through contact with viremic animal fluids, or through the bite of an infected mosquito. Following the large RVF outbreak in East Africa in 2006–2007, several outbreaks were reported in the Comoros Archipelagos and Madagascar. Thus, in Mayotte Island, human cases were first reported in 2007–2008, and later in 2018–2019^6^. In La Reunion, *Cx. quinquefasciatus* might ensure local transmission of this virus, as in Kenya^7^. Other zoonotic arboviroses transmitted from birds to mammals (horses, pigs) and humans by *Culex* mosquitoes are already present in the region, such as West Nile^8^. Still others are at risk of introduction, such as Japanese encephalitis. Research is needed to better understand the vector risk of mosquito-borne human and zoonotic viral diseases in SWIO and to improve their surveillance and control.

Control of *Aedes* vectors is challenging because of their multiple, small breeding sites. Current control methods, particularly insecticide spraying, are not effective, thus justifying research for innovative control methods. Among these methods, the Sterile Insect Technique (SIT) is promising^9^. This environment-friendly technique involves the large-scale release of sterile male mosquitoes to reduce mosquito populations. Mosquitoes are mass-reared in the laboratory, sexed, and then sterilized through irradiation before being released^10^. Wild virgin females mate with sterile males and have no offspring. Several trials have been implemented in La Reunion, among which the SIT is being tested against *Ae. albopictus*^11^ and a derived strategy, the boosted SIT, whereby sterile males are also used as carriers of a biocide towards females and larval sites, against *Ae. aegypti*^12^.

For an SIT trial to be successful, it is essential to have accurate information on the target population, its characteristics and phenology before starting any control activity. This is all the more important in La Reunion as the local population of *Ae. aegypti*—targeted in the boosted SIT trials, is peculiar with respect to populations found elsewhere^13^, and co-occurs with *Ae. albopictus* mosquitoes. A crucial parameter to monitor during SIT control trials is the ratio of released sterile males insects to wild males^14^. Therefore, the sensitivity of the trapping method for males is an important feature.

One of the most commonly used traps for monitoring *Aedes* mosquitoes is the BG-Sentinel trap (Biogents, Germany). This trap is usually baited with BG-Lure (Biogents, Germany)—a mixture of lactic acid, ammonia and caproic acid—mimicking the odor of human skin, to increase the catches^15^. The addition of carbon dioxide (CO_2_) is also recommended by the trap manufacturer to increase catches^16^. However, variable efficacy for the possible trapping strategies—combinations of these two attractants, has been reported for the two populations of *Aedes* species present in La Reunion.

The goal of this study was to identify the best trapping strategy for catching adult *Ae. aegypti*, with a particular focus on males, as a baseline data collection for reference in a subsequent boosted SIT trial. We also included data on *Ae. albopictus* and *Cx. quinquefasciatus*.

## Results

In this document, the detection probability is the probability to detect at least one mosquito of a given species during a 24-h trapping session, given the known presence of this species in the study area. The apparent density is the mean number of individuals of given species and sex caught during a 24-h trapping session.

Overall, 951 *Ae. aegypti*, 618 *Ae. albopictus* and 737 *Cx. quinquefasciatus* were identified out of a total of 2306 catches. On a few occasions, one of the traps failed (lack of CO_2_, or defective battery). The design was therefore slightly unbalanced. However, the missing data were independent of the response, and the maximum-likelihood estimates of the model coefficients were therefore unbiased. In total, results from 89 trapping sessions were available, whereas the expected total was 96 (92.7%).

### Observations

#### Detection probability (Fig. 1, Table S1)

**Figure 1:**
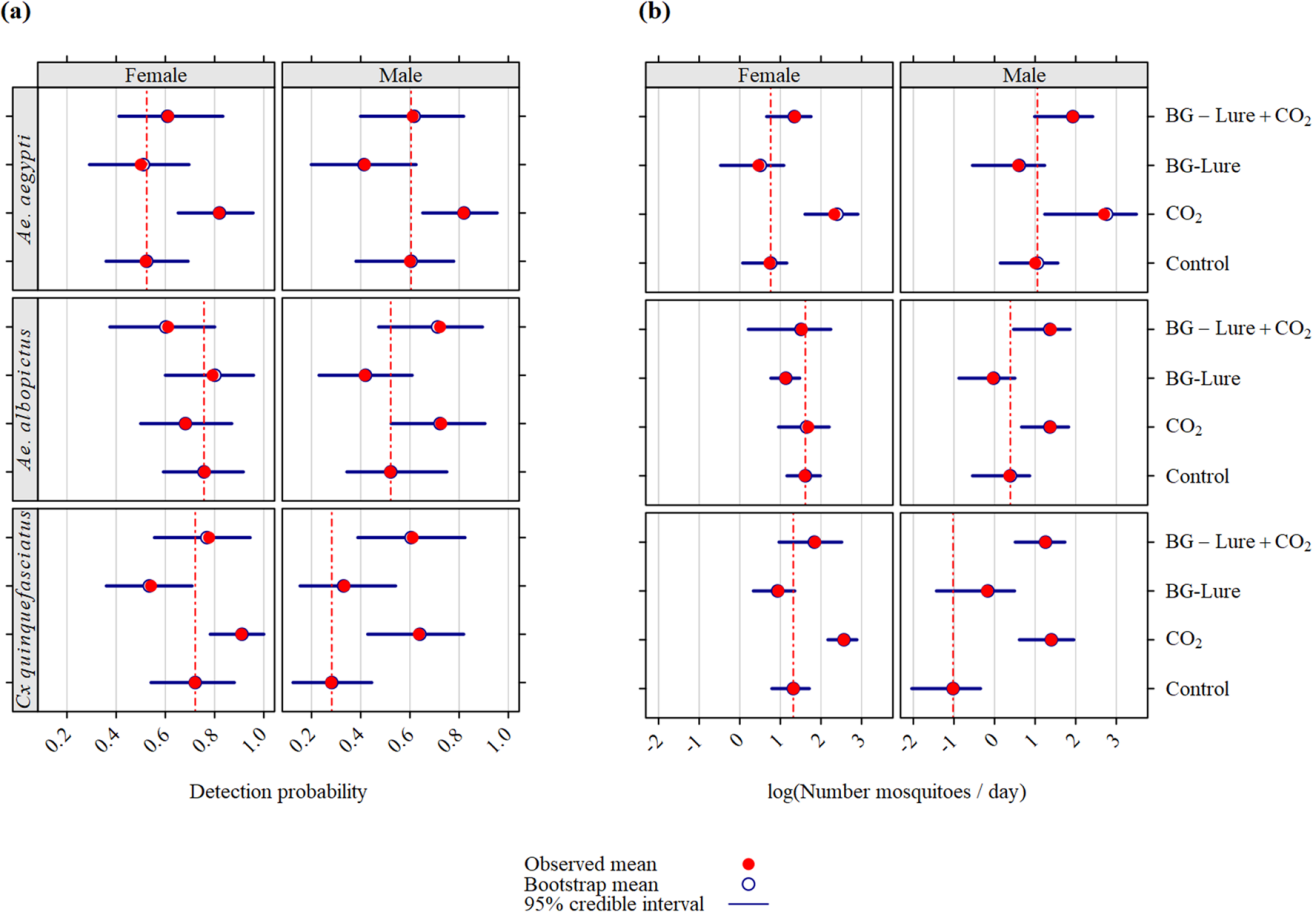
Observed detection probability (a) and apparent density (b) for well-established adult *Aedes aegypti*, *Ae. albopictus*, and *Culex quinquefasciatus* populations in two study sites located in La Reunion.

Similar response patterns were observed for male and female *Ae. aegypti* mosquitoes.

- For males, the highest detection probability P_d_ was observed for the CO_2_-baited trap (P_d_ = 0.82, 95% credible interval [0.65; 0.95]), and the lowest for the BG-Lure-baited trap (P_d_ = 0.42 [0.20; 0.62]).
- For females, the highest detection probability was also observed for the CO_2_-baited traps (P_d_ = 0.82 [0.65; 0.96]), and the lowest for the BG-Lure-baited trap (P_d_ = 0.50 [0.29; 0.70]).

Thus, baiting traps with CO_2_ provided a higher detection probability for *Ae. aegypti*. Moreover, there was no advantage to using BG-Lure—either alone, or in association with CO_2_.

For *Ae. albopictus*, the detection probability pattern according to the trapping strategy was quite different for females and males.

- For males, adding CO_2_ increased the detection probability with respect to the control category (P_d_ = 0.73 [0.52; 0.90] vs. P_d_ = 0.52 [0.34; 0.75]). The addition of BG-Lure did not alter the detection probability, irrespective of CO_2_ addition.
- For females, the detection probability was much higher than for males (P_d_ = 0.76 [0.59; 0.92]) for the control category. The addition of BG-Lure was associated with a slight not significant, increase in the detection probability (P_d_ = 0.79 [0.60; 0.96]). The addition of CO_2_ was associated with a decreased detection probability (P_d_ = 0.68 [0.50; 0.87]), and decrease was even stronger when CO_2_ was combined with BG-Lure (pa = 0.61 [0.37; 0.80]).

Thus, for this species, the addition of CO_2_ greatly improved the detection probability for males, while it decreased it for females. In both sexes, the addition of BG-Lure did not improve this probability compared with the use of CO_2_.

Regarding *Cx. quinquefasciatus*, the detection probability was much lower in males than in females for the control category (P_d_ = 0.28 [0.12; 0.44] vs. 0.72 [0.54; 0.88]). The addition of CO2 increased the detection probability in both males and females (Pd = 0.64 [0.43; 0.82] and 0.96 [0.91; 1.00]). Conversely, the addition of BG-Lure, alone or in combination with CO_2_, did not increase the detection probability.

#### Apparent density (Fig. 1 and Table S2)

For *Ae. aegypti*, the addition of CO_2_ was associated with a strong increase in apparent density D_a_, compared with the control trapping strategy in males (D_a_ = 14.9 [3.4; 32.9] vs. 2.7 [1.2; 4.8]), and females (D_a_ = 10.3 [5.0; 18.4] vs. 2.1 [1.1; 3.2]).

Conversely, the addition of BG-Lure was associated with a decreased apparent density compared with the control and CO_2_ trapping strategies in both males (D_a_ = 1.8 [0.6; 3.4] and 6.9 [2.7; 11.2]), and females (D_a_ = 1.6 [0.6; 3.0] and 3.9 [1.9; 5.8]).

For *Ae. albopictus*, the addition of CO_2_ was associated with an increased apparent density compared to the control trapping strategy in males (D_a_ = 4.0 [1.9; 6.2] vs. 1.4 [0.6; 2.4]). In females, the addition of CO_2_ did not alter the apparent density (D_a_ = 5.4 [2.6; 9.0]). The addition of BG-Lure was associated with a decreased apparent density with respect to the control and CO_2_ trapping strategies in both males (D_a_ = 1.0 [0.4; 1.7] and 4.0 [1.6; 6.4]) and females (D_a_ = 3.1 [2.2; 4.4] and 4.6 [1.2; 9.4]).

For *Cx. quinquefasciatus*, the addition of CO_2_ was associated with a strong increase in apparent density compared with the control trapping strategy in both males (D_a_ = 4.0 [1.8; 7.0] vs. 0.4 [0.1; 0.7]) and females (D_a_ = 13.0 [8.8; 17.9] vs. 3.8 [2.2; 5.5]). Conversely, the addition of BG-Lure was associated with a decreased apparent density, compared with the control and CO_2_ trapping strategies, both males (D_a_ = 0.8 [0.2; 1.6] and 3.5 [1.7; 5.7]) and females (D_a_ = 2.6 [1.4; 3.9] and 6.4 [2.6; 12.3]).

### Multi-model averaging: fitted apparent density

The coefficients of the multi-model averaged (MMA) models of apparent density for *Ae. aegypti*, *Ae. albopictus*, and *Cx. Quinquefasciatus*—and their 95% CI, are shown in supplementary-information Tables S3, S4 and S5.

Because the interpretation of single coefficients is difficult with the MMA approach, we used more global indicators to assess statistical significance and the bait effect on apparent mosquito density.

**Figure 2:**
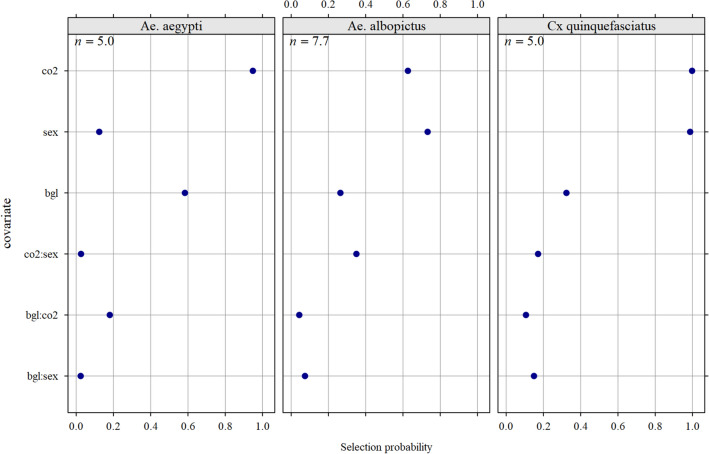
Average probability (200 replicates) for a covariate to be included in the model subset kept for multi hurdle-model averaging of adult mosquito apparent density fitted on data collected in 2020 during a field trial (89 trapping sessions) in La Reunion.

The same covariates were used for selection in the three species apparent density models: three main effects (CO_2_, BG-Lure, and sex), and their two-way interactions. The importance of each covariate (i.e., its selection frequency in each bootstrapped model) is shown on Fig. 2.

- For *Ae. aegypti*, CO_2_ was the most important covariate, followed by BG-Lure. However, the effect of BG-Lure was opposite to that of CO_2_. Sex had a minor importance, with similar response patterns by bait category, in males and females. No strong bias of sex-ratio was thus observed, both in unbaited and CO2 baited traps (Fig. S1).
- For *Ae. albopictus*, sex was the most important covariate, closely followed by CO_2_. In addition, the interaction between sex and CO_2_ was selected in 30% of the models. The importance of BG-Lure was similar to this interaction. However, it increased the detection probability, and lowered the apparent density.
- For *Cx. quinquefasciatus*, CO_2_ and sex were equally and strongly important: differences in response were significant as a function of sex (apparent density was higher in females than in males) and CO_2_ (apparent density was higher with CO_2_-baited traps). The BG-Lure was consistently associated with a slight reduction in apparent density, regardless of whether the trap was baited with CO_2_.

**Figure 3:**
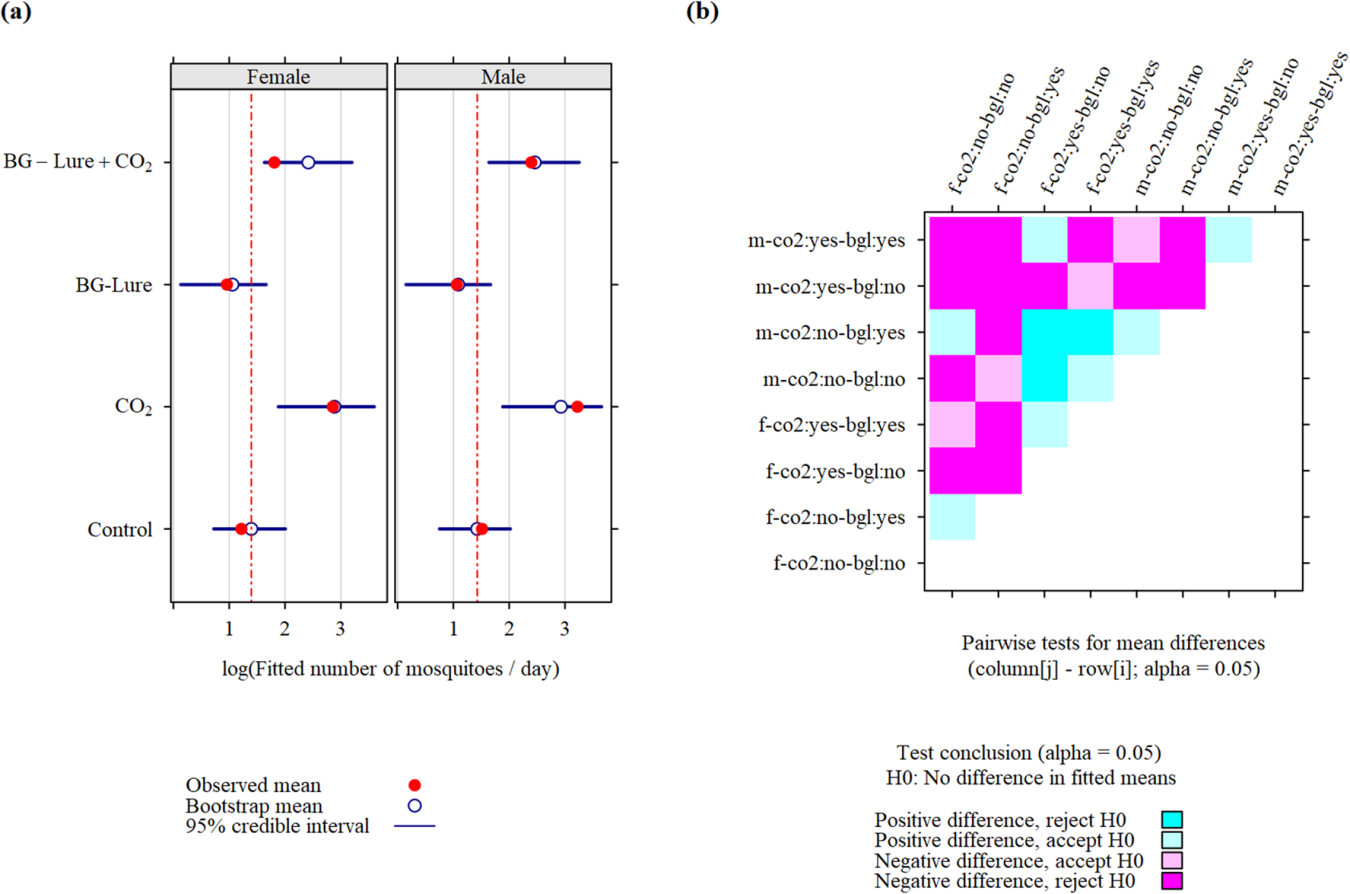
Fitted apparent density (**a**) and corresponding pairwise tests (**b**) for differences in fitted means for adult *Aedes aegypti* mosquitoes collected in 2020 (89 trapping sessions) during a field experiment in La Reunion. Coding scheme for row and column labels: sex (m/f)—CO2 (co2:yes/co2:no)—BG-Lure (bgl:yes/bgl:no).

**Figure 4:**
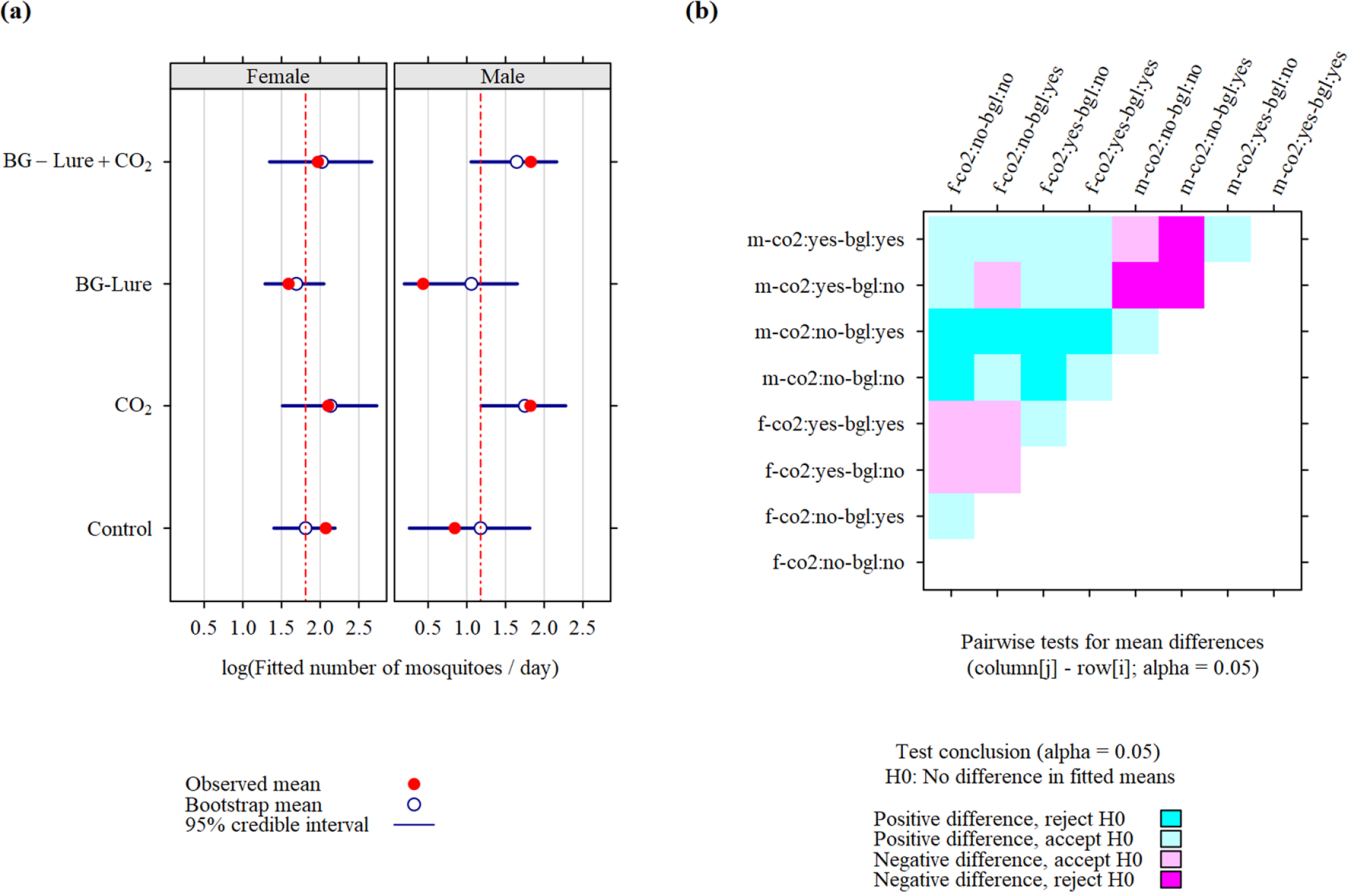
Fitted apparent density (**a**) and corresponding pairwise tests (**b**) for differences in fitted means for adult *Aedes albopictus* mosquitoes collected in 2020 (89 trapping sessions) during a field experiment in La Reunion. Coding scheme for row and column labels: sex (m/f)—CO2 (co2:yes/co2:no)—BG-Lure (bgl:yes/bgl:no).

**Figure 5:**
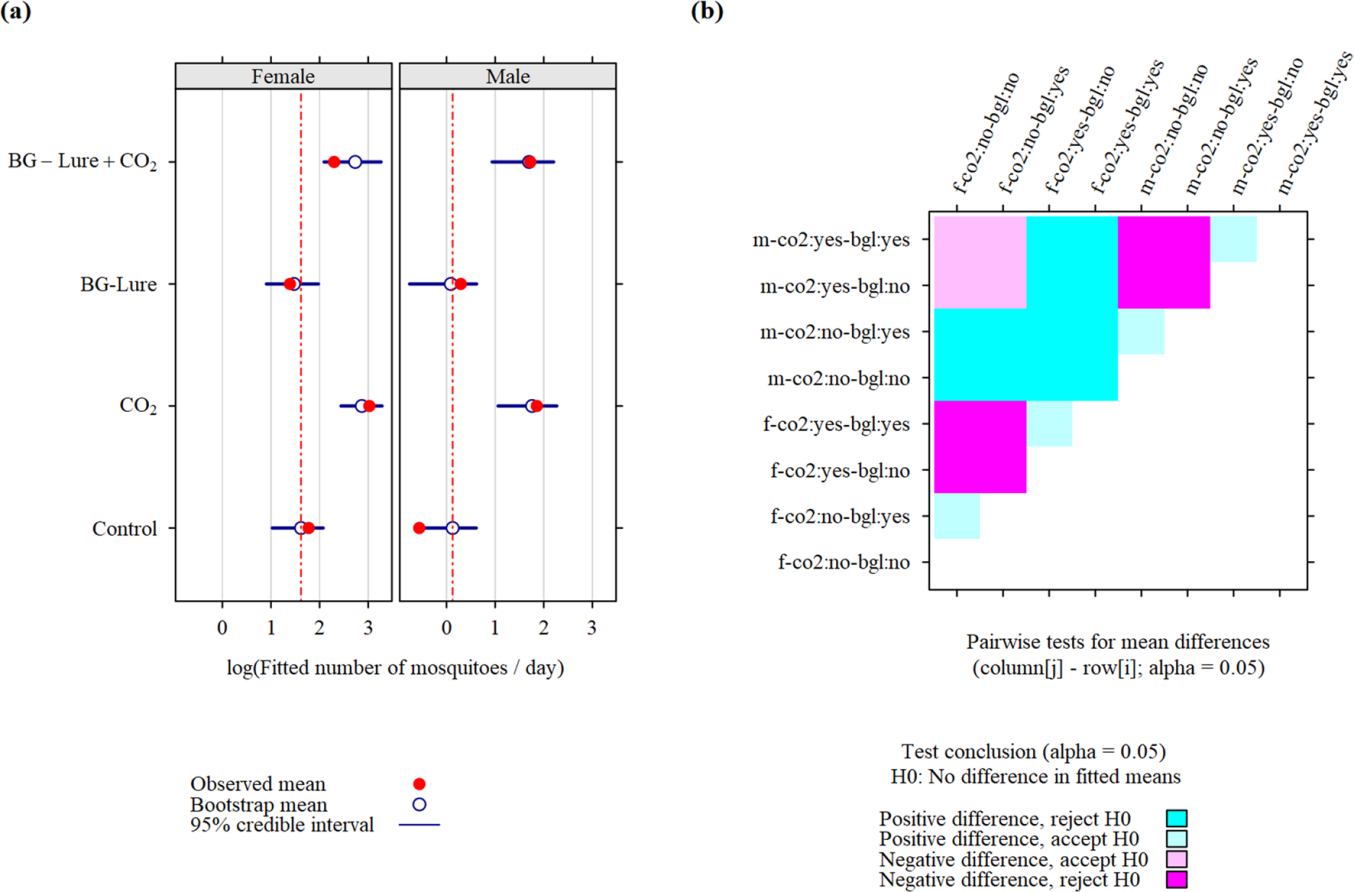
Fitted apparent density (**a**) and corresponding pairwise tests (**b**) for differences in fitted means for adult *Culex quinquefasciatus* mosquitoes collected in 2020 (89 trapping sessions) during a field experiment in La Reunion. Coding scheme for row and column labels: sex (m/f)—CO2 (co2:yes/co2:no)—BG-Lure (bgl:yes/bgl:no).

The observed and fitted apparent density (Fig. 3, Fig. 4, and Fig. 5) were generally close, with exceptions for the trapping strategies BG-Lure + CO_2_, and CO_2_, mainly for *Ae. aegypti*, and *Cx. quinquefasciatus*. Because these data did not show a positive effect of BG-Lure in increasing mosquito apparent density, this lack of fit did not change the conclusions with respect to the question asked in this study (to bait or not to bait adult mosquito traps):

- In males and females of each species, baiting the traps with CO_2_ alone increased the apparent density with respect to the control scheme. However, this increase was not statistically significant for female *Ae. albopictus* (P > 0.05).
- For *Ae. aegypti* and *Cx. quinquefasciatus*, the addition of BG-Lure alone was associated with a decreased apparent density, but this decrease was only significant for *Cx. quinquefasciatus* (P < 0.05). In *Ae. albopictus*, a non-significant decrease in density was observed.
- The apparent density was lower when BG-Lure was used alone than when it was used together with CO_2_, but it was not significant (P > 0.05) in *Ae. albopictus*.

Thus, baiting the traps with CO_2_ generally increased the apparent density. The same effect was not observed with BG-Lure. The combination of the two baits did not improve the effect of CO_2_ alone.

## Discussion

This study is part of a research project to control an isolated population of *Ae. aegypti* mosquitoes in a target area of St Joseph using boosted SIT^12^. It was therefore important to identify the best way to catch *Ae. aegypti* mosquitoes to monitor the target population by assessing its abundance as reliably as possible. We found the frequency and density of trap catches were much higher with CO_2_ for *Ae. aegypti* mosquitoes and the other two populations of *Ae. albopictus* and *Cx. quinquefasciatus* mosquitoes. The use of this bait alone also allowed catching large numbers of males, which is crucial for monitoring the impact of SIT. Actually, the ratio of sterile to wild males is a key indicator during SIT programs^14^, and together with the induced sterility in eggs, this ratio allows monitoring the competitiveness of sterile males^14^. This parameter is crucial for the prediction of the efficiency of both SIT and, to a lesser extent boosted SIT trials^17^.

Conversely, catches were lower when CO_2_ was combined with BG-Lure. Traps with BG-Lure alone showed null or negative effects on all species compared to control traps. Therefore, sampling with BG-Lure underestimated the actual density of *Ae. aegypti*, *Ae. albopictus*, and *Cx. quinquefasciatus*, unlike CO_2_, which gave a better estimate of the relative abundance of mosquitoes of these three species in the study areas. These results are surprising considering the use of BG-Lure has often been considered the most efficient way to catch them^18^. Other studies have found that baiting BG-Sentinel traps with BG-Lure coupled with CO_2_ was the most effective way to catch adults of the three mosquito species studied here^19^. However, some studies have found that BG-Sentinel traps baited with CO_2_ alone were able to catch significantly more individuals than BG-Sentinel traps baited with BG-Lure—coupled or not with CO_2_^20^. Also, Staunton et al*.*^21^ did not observe any impact of BG-Lure on the catches of *Ae. aegypti* and *Ae. albopictus* mosquitoes, both with Male Aedes Sound Traps (MASTs) and BG-Sentinel traps in Cairns, Australia and Madang, Papua New Guinea. Visser et al*.*^22^ found BG-Sentinel traps baited with CO_2_ coupled with the MB5 blend—another blend also mimicking human body odor, caught more *Ae. aegypti* and *Cx. quinquefasciatus* than traps either baited with MB5 blend, or CO_2_ alone. These authors also reported they obtained significantly more catches baiting the BG-Sentinel traps with the MB5 blend than with the BG-Lure. In our case, baiting the BG-Sentinel traps with CO_2_ alone caught more individuals but it might be interesting to assess its effect in combination with the MB5 blend. Baiting BG-Sentinel traps with hexanoic acid and natural human odors improved catches of *Ae. aegypti* with regard to BG-Lure alone, the latter including additional volatile components not present in BG-Lure^23^.

Several methods of CO_2_ release are possible. We used CO_2_ bottles but it is also possible to use dry ice, although flow control is much better with pressurized bottles. Visser et al*.*^22^ used CO_2_ produced with yeast fermented sugar and they also obtained better catches. Again, the latter method is not as accurate in terms of flow control. We used a low throughput (0.2 L/24 h) to optimize the trapping costs because we observed, during preliminary trials, that this was enough to attract mosquitoes. Higher rates of 2 kg/24 h (using dry ice) were previously reported^24^. These authors also reported that three mice had the same attractiveness as CO_2_ at this rate. Considering a mean body mass of 20 g and a maximum expelled volume of CO_2_ of 95.5 $\pm$ 15.4 mL/kg/min^25^, a mouse should expel $\simeq$ 1.9 mL/min which is close to 2.8 kg/24 h, i.e., much more than what we used here. One of the reasons that might explain this efficiency at such a low release rate is that CO_2_ was released inside the BG-Sentinel trap (contrary to manufacturer’s recommendations). Thus, an increased concentration of CO_2_ was found in the trap, with a decreasing gradient outside the trap, thanks to the holes made in the cover. This modified device setting might better mimick a real host.

The lack of bias in the *Ae. aegypti* sex-ratio estimated with CO_2_-baited BG-Sentinel traps was also reported in mark–release–recapture experiments implemented with *Ae. albopictus* mosquitoes in La Reunion^26^. Thus, these CO_2_-baited traps might be used to monitor *Ae. aegypti* populations without biasing the estimated abundance of both sexes. Females are attracted by CO_2_ which activates their host-seeking behavior^27^. Male mosquitoes, like females, have receptor neurons that allow them to detect CO_2_^28^. These receptors help them finding hosts for female blood meals, and thus locating areas where females are abundant. Bohbot et al*.*^29^ also reported *Ae. aegypti* males respond to host odors.

In addition, *Ae. aegypti* mosquitoes may form swarms and mate during the day near hosts^30^. These swarms are primarily composed of males: females are attracted by the pheromons and sounds they emit^31^. Traps with CO_2_ can then attract males and females present in the swarm.

In our observations, BG-Lure had a negative effect on the frequency and apparent density of *Ae. aegypti* mosquitoes. Either the BG-Lure prevented mosquitoes from correctly detecting CO_2_, or it repelled mosquitoes.

In *Ae. albopictus* mosquitoes, our results showed a bias in the sex-ratio of *Ae. albopictus*, with 3.5 times more females than males caught, in agreement with several other studies conducted on this species in La Reunion^32^. Le Goff et al*.*^24^ reported baiting the BG-Sentinel traps with CO_2_ coupled with mice increased catch density, with catch density proportional to the density of mice in the bait. The attractiveness of mice was related to the CO_2_ or body heat, and not to body odor.

Although fewer males were caught than females, we still found a significant increase in male *Ae. albopictus* catches when the trap contained CO_2_ coupled with the BG-Lure, as observed by Pombi et al*.*^33^. Catches of males with CO_2_ alone were significantly higher than those in the control traps. In contrast to previous observations for *Ae. aegypti* and *Cx. quinquefasciatus*^22^, Pombi et al*.*^33^ also reported that baiting BG-Sentinel traps with MB5 blend was as efficient as baiting them with the BG-Lure for *Ae. albopictus*. Cilek et al*.*^34^ found no significant difference in the apparent density of *Ae. albopictus* and *Cx. quinquefasciatus* for traps baited with CO_2_ or BG-Lure. They also reported a higher apparent female density for traps baited with CO_2_, compared with traps baited with BG-Lure plus octenol and CO_2_.

In this study, BG-Sentinel CO_2_-baited traps were more efficient in catching *Ae. aegypti*, *Ae. albopictus*, and *Cx. quinquefasciatus* than the same traps with other trapping strategies—thus highlighting the necessity to assess the most effective trapping methods in the local context of mosquito monitoring programs.

In addition, this study provided new insights on the ecology of local *Ae. aegypti* and *Ae. albopictus* populations, and allowed improving the monitoring and management of these populations—notably for *Ae. aegypti* mosquitoes whose population was considered as scarce^35^. An update of its distribution and abundance using a more sensitive trapping method would be useful to carry out at the island scale.

Finally, the risk of competitive replacement has been pointed out by expert groups^36^, in case of targeting only one of the two specific mosquito populations by a control technique such as the SIT. These two populations are in competition in our study area, where *Ae. aegypti* population even developed a resistance to satyrization^37^. The identification of such a risk requires the use of traps with similar efficiency for the competing populations.

In conclusion, we decided to use BG sentinel traps baited with CO_2_ 0.2 L/24 h to monitor the impact of the boosted SIT trial implemented in Saint-Joseph.

## Methods

### Field sites and study population

A field experiment was implemented from 5 October to 12 November 2020 on two study sites in Saint-Joseph, a municipality located in the south of La Reunion. The first study site (21°23′04.2″S 55°38′45.3″E) was a 10-ha orchard covered with vacoa trees (*Pandanus edulis*), palm and other fruit trees located next to the Langevin River outfall. It was surrounded by a school, a library, and houses. The second study site (21°22′48.2″S 55°39′34.7″E) was a 350 m long and 40 m wide isolated ravine, ending in Indian Ocean. Both study sites had well-established mosquito populations of *Ae. aegypti*, *Ae. albopictus*, and *Cx. quinquefasciatus*.

### Study design and data collection

BG-Sentinel traps (Biogents, Germany) consist of a collapsible, dark blue fabric container and a white lid with holes covering its opening. Air is sucked into the trap through a black catch pipe by an electrical fan, through a black netting acting as a cage. A Latin square design was used to assess the effect of two baits on BG-Sentinel traps attractiveness: carbon dioxide (CO_2_) and BG-Lure (Biogents, Germany). BG-Lure was used as specified by the manufacturer: each packaging was opened at the beginning of the trial and used for a total duration of less than 1.5 months whereas the manufacturer claims an efficiency of up to 5 months (https://sea.biogents.com/attractants/bg-lure-attractant/, accessed on 18th October 2022).

**Figure 6:**
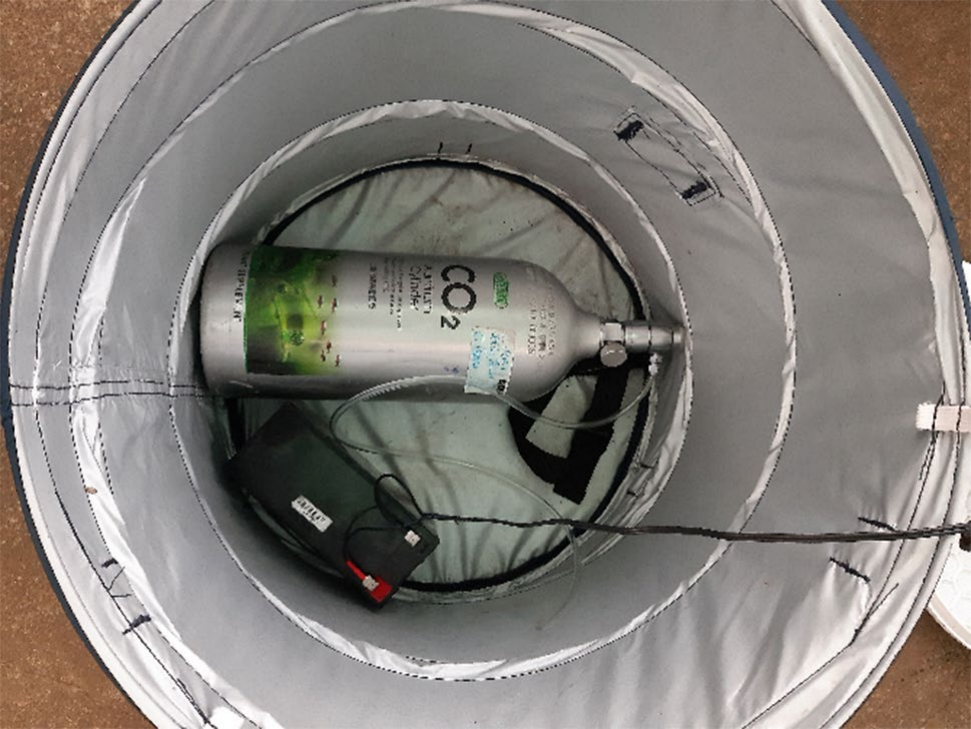
Inside view of a BG-Sentinel trap containing a battery and a CO_2_ bottle.

BG-Sentinel traps were baited with one of the four trapping strategies: (i) BG-Lure, (ii) CO_2_ 0.2 L/24 h, (iii) both, and (iv) no bait. To release CO_2_, bottles used in fish-keeping (ISTA CO_2_ Aluminum Cylinder 2.0L FACE SIDE or FACE UP-Plants Growth Supplement | Aquatic Plant Fertilizer (2 L)) were used (Fig. 6). The flow rate was measured by dipping a tube attached to the outlet of the CO_2_ bottle into the water and counting the number of bubbles over a given time, in this case 30 bubbles over 10 s, i.e. 0.2 L/24 h.

Each trapping strategy was randomly allocated to a trap at each 24 h trapping session, thus covering the entire daily mosquito activity cycle. There were four traps per site per trapping session, i.e., eight trapping results for each session. Four trapping sessions were required to complete a block (each trapping strategy being assigned to each trap), i.e., a total of 32 trapping results/block. Three replicates of this block were implemented in October and November 2020 (early rainy season), thus providing a total of 96 trapping results (12 results/trap). The environmental effects controlled by the Latin square design were trapping date (proxy for air temperature, relative humidity, wind speed and direction) and trapping site (proxy for habitat type, distance to breeding and resting sites).

At the end of each 24-h trapping session, mosquitoes were collected from each BG-Sentinel trap and frozen at − 20 °C to kill them. They were then poured into Petri dishes for identification using a binocular and a morphological key adapted to local mosquitoes^2^. Individuals of *Ae. aegypti*, *Ae. albopictus*, and *Cx. quinquefasciatus* were counted by species and sex. Data were entered and stored in a relational database management system.

### Data analysis

Separate analyses were done for each mosquito species. To assess the effects of trapping strategies, we chose a hurdle model jointly accounting for the detection probability of the mosquitoes, and their apparent density^38^. This model was made of two sub-models which were jointly fitted: (i) a logistic Bernoulli model for the presence data (detection probability), and a zero-truncated negative binomial model for the apparent-density counts. The same covariates were used to model the detection probability and the apparent density.

The latter was chosen to account for the large variance met in this dataset—related to the peculiarities of *Aedes* mosquitoes ecology. Indeed, males of *Ae. aegypti* sometimes aggregate in swarms of thousands of insects, triggered by aggregation pheromones^39^. Females are also attracted by these swarms, as well as mosquitoes from other species. When such a swarm met a trap, hundreds of mosquitoes were caught during a single trapping session. This happened several times during the experiment. We did not discard the corresponding data because they are an important feature of *Aedes* ecology. Therefore, we did not expect to select a single best model adapted to an unrealistic average situation.

Instead, we adopted a multi-model averaging approach, well adapted when there is not a single plausible model with respect to the available data^40^. A “full” model was defined, representing the most complex structure expected in the dataset. From this full model, a set of nested, plausible sub-models was defined, and all these models were fitted using a maximum-likelihood method. They were ranked using AICc, the small-sample version of the Akaike information criterion. The AICc difference between consecutive models was computed, and divided by the AICc difference between the first and last models. This quantity is the Akaike weight. It was used as a relative model plausibility indicator, and to compute weighted means of the fitted values from each compared model, i.e., multi-model averaging (MMA). We applied the approach on each bootstrapped dataset to compute 95% credible intervals for the MMA-fitted values.

To estimate the 95% credible intervals of observed and fitted values, we used a bootstrap resampling procedure of the observed dataset^41^. The combination of sites and dates (two sites, four dates by replicate, and three replicates) was taken as the 24 resampling units of four trapping sessions each (i.e., the four dates). A random sample of these units was drawn with replacement in the initial data set, with the same size in sampling units. It was used to compute the simulated means. The process was iterated 200 times. The 2.5% and 97.5% quantiles of the empirical distributions of each simulated mean were used as the 95% credible interval of population means.

The R software and computing environment^42^ was used for data analysis, in particular statistical packages pscl for hurdle models^38^, and MuMIn for multi-model averaging and inference^43^, implementing the methods described in Burnham & Anderson^44^. Throughout this document, $\alpha$ was set to 0.05.

## Supplementary Information

Supplementary Information 1.

Supplementary Information 2.

## Data Availability

Data and R code needed to run the analysis are available in a master R Markdown document^45^ named bgtrap.Rmd containing the dataset—as an R object named tab, and the master R code. Most of the work is done by R functions automatically sourced in the running R session from an external file called functions.R. The list of add-on R packages needed to run the analysis, as well as some code to install—and possibly update them, are available in an R file called packages.R, also automatically sourced in the R session. All these files are available in the section Supplementary Information containing further instructions.
