# Supplementary material for: Optimization of adult mosquito trap settings to monitor populations of Aedes and Culex mosquitoes, vectors of arboviruses in La Reunion
Source: Sci Rep. 2022 Nov 15;12:19544. doi: 10.1038/s41598-022-24191-9 (PMC9666360; doi:10.1038/s41598-022-24191-9)
Supplement: Supplementary file 2 — Supplementary Information 2. [file 41598_2022_24191_MOESM2_ESM.docx]

Optimization of adult mosquito trap settings to monitor populations of *Aedes* and *Culex* mosquitoes, vectors of arboviruses in La Reunion

Supplementary information

Iris Claudel^1,a^, Ronan Brouazin^1,a^, Renaud Lancelot^2,3^, Louis-Clément Gouagna^4^, Marlène Dupraz^2,3^, Thierry Baldet^2,3^, Jérémy Bouyer^2,5^*

^1^ UMR Mivegec (Maladies Infectieuses et Vecteurs: Écologie, Génétique, Évolution et Contrôle), IRD-CNRS-Univ. Montpellier, 97410 Saint-Pierre, La Réunion

^2^ UMR Astre (Animals, Health, Territories, Risks, Ecosystems), Cirad, Inrae, Univ. Montpellier, 34398 Montpellier, France

^3^ Cirad, UMR Astre, 97491 Sainte Clotilde, La Réunion

^4^ UMR Mivegec, 34394 Montpellier, France

^5^ Insect Pest Control Laboratory, Joint FAO/IAEA Programme of Nuclear Techniques in Food and Agriculture, IAEA Vienna, Wagramer Strasse 5, 1400 Vienna, Austria

^a^ These authors contributed equally to the work

(*) Corresponding author: Jérémy Bouyer [jeremy.bouyer@cirad.fr](mailto:jeremy.bouyer@cirad.fr)

# 1 Figure


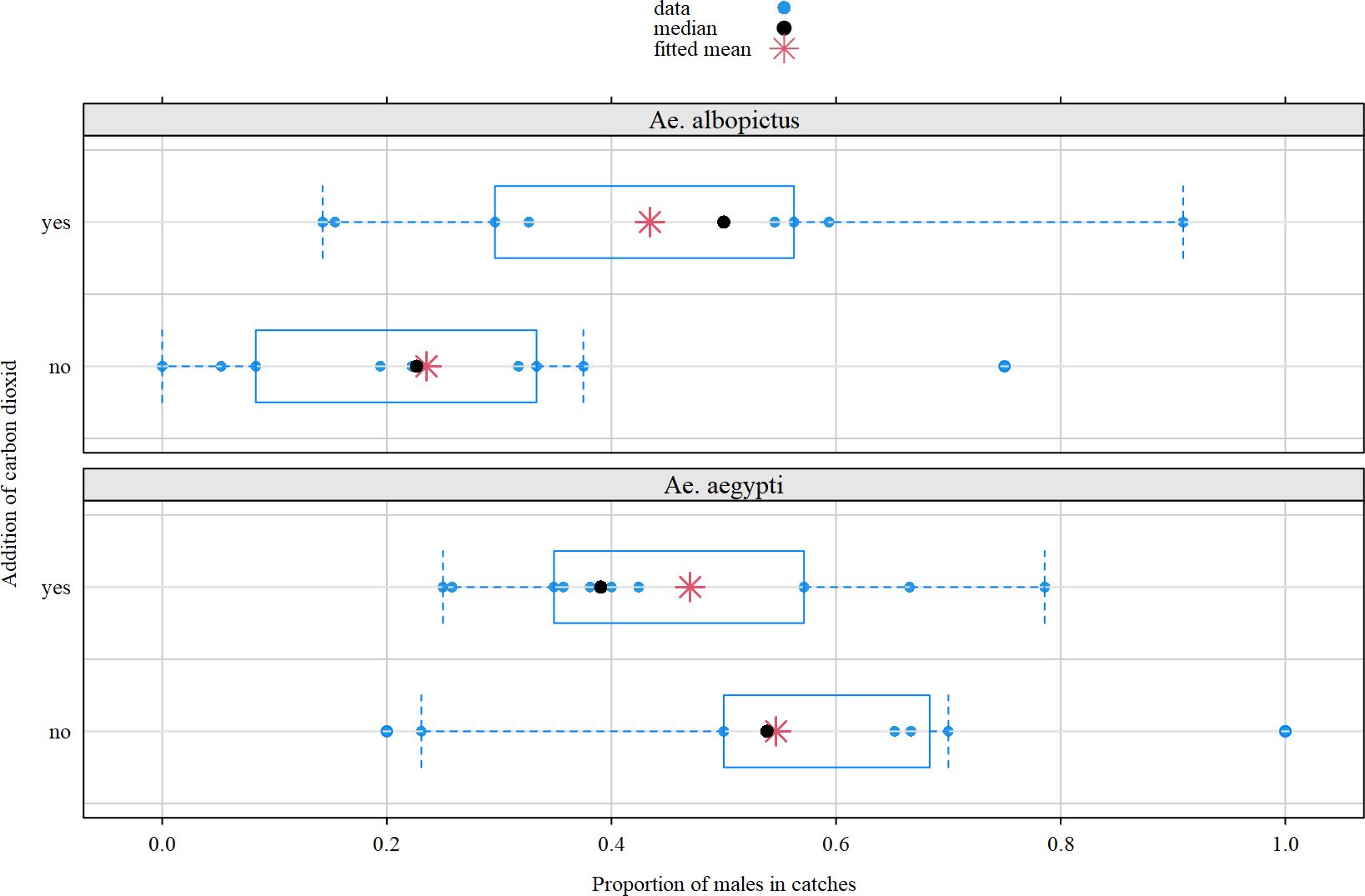


Fig. S1. Proportion of male mosquitoes in catches, according to species and CO_2_-baiting status of BG-Sentinel traps during a field experiment implemented in 2020, La Reunion. Data from BG-Lure-baited traps were excluded; means were fitted with a beta-binomial logistic regression model.

# 2 Tables

Tab. S1. Detection probability of adult mosquitoes obtained from data collected in 2020 (89 trapping sessions) during a field experiment in La Reunion.

|  | | Bootstrap estimates (B = 200) | | |
| --- | --- | --- | --- | --- |
| Bait | Observed | Estimated | Lower 95% limit | Upper 95% limit |
| Ae. Aegypti | | | | |
| *Males* | | | | |
| Control | 0.60 | 0.60 | 0.38 | 0.78 |
| CO_2_ | 0.82 | 0.82 | 0.65 | 0.95 |
| BG-Lure | 0.42 | 0.41 | 0.20 | 0.62 |
| CO_2_ + BG-Lure | 0.61 | 0.62 | 0.40 | 0.82 |
| *Females* | | | | |
| Control | 0.52 | 0.52 | 0.36 | 0.69 |
| CO_2_ | 0.82 | 0.82 | 0.65 | 0.96 |
| BG-Lure | 0.50 | 0.51 | 0.29 | 0.70 |
| CO_2_ + BG-Lure | 0.61 | 0.61 | 0.41 | 0.83 |
| Ae. Albopictus | | | | |
| *Males* | | | | |
| Control | 0.52 | 0.52 | 0.34 | 0.75 |
| CO_2_ | 0.73 | 0.72 | 0.52 | 0.90 |
| BG-Lure | 0.42 | 0.42 | 0.23 | 0.61 |
| CO_2_ + BG-Lure | 0.72 | 0.71 | 0.47 | 0.89 |
| *Females* | | | | |
| Control | 0.76 | 0.76 | 0.59 | 0.92 |
| CO_2_ | 0.68 | 0.68 | 0.50 | 0.87 |
| BG-Lure | 0.79 | 0.80 | 0.60 | 0.96 |
| CO_2_ + BG-Lure | 0.61 | 0.60 | 0.37 | 0.80 |
| Cx. Quinquefasciatus | | | | |
| *Males* | | | | |
| Control | 0.28 | 0.28 | 0.12 | 0.44 |
| CO_2_ | 0.64 | 0.64 | 0.43 | 0.82 |
| BG-Lure | 0.33 | 0.33 | 0.15 | 0.54 |
| CO_2_ + BG-Lure | 0.61 | 0.60 | 0.39 | 0.82 |
| *females* | | | | |
| Control | 0.72 | 0.72 | 0.54 | 0.88 |
| CO_2_ | 0.91 | 0.91 | 0.78 | 1.00 |
| BG-Lure | 0.54 | 0.53 | 0.36 | 0.71 |
| CO_2_ + BG-Lure | 0.78 | 0.77 | 0.56 | 0.94 |

Tab. S2. Apparent density of adult mosquitoes obtained from data collected in 2020 (89 trapping sessions) during a field experiment in La Reunion.

|  | | | | |
| --- | --- | --- | --- | --- |
|  | | Bootstrap estimates (B = 200) | | |
| Bait | Observed | Estimated | Lower 95% limit | Upper 95% limit |
| *Ae. Aegypti* | | | | |
| Males | | | | |
| Control | 2.7 | 2.9 | 1.2 | 4.8 |
| CO_2_ | 14.9 | 15.8 | 3.4 | 32.9 |
| BG-Lure | 1.8 | 1.8 | 0.6 | 3.4 |
| CO_2_ + BG-Lure | 6.9 | 6.9 | 2.7 | 11.2 |
| Females | | | | |
| Control | 2.1 | 2.1 | 1.1 | 3.2 |
| CO_2_ | 10.3 | 11.0 | 5.0 | 18.4 |
| BG-Lure | 1.6 | 1.7 | 0.6 | 3.0 |
| CO_2_ + BG-Lure | 3.9 | 3.8 | 1.9 | 5.8 |
| *Ae. Albopictus* | | | | |
| Males | | | | |
| Control | 1.4 | 1.5 | 0.6 | 2.4 |
| CO_2_ | 4.0 | 3.9 | 1.9 | 6.2 |
| BG-Lure | 1.0 | 1.0 | 0.4 | 1.7 |
| CO_2_ + BG-Lure | 4.0 | 3.9 | 1.6 | 6.4 |
| Females | | | | |
| Control | 5.0 | 5.0 | 3.2 | 7.3 |
| CO_2_ | 5.4 | 5.2 | 2.6 | 9.0 |
| BG-Lure | 3.1 | 3.1 | 2.2 | 4.4 |
| CO_2_ + BG-Lure | 4.6 | 4.5 | 1.2 | 9.4 |
| *Cx quinquefasciatus* | | | | |
| Males | | | | |
| Control | 0.4 | 0.4 | 0.1 | 0.7 |
| CO_2_ | 4.0 | 4.1 | 1.8 | 7.0 |
| BG-Lure | 0.8 | 0.8 | 0.2 | 1.6 |
| CO_2_ + BG-Lure | 3.5 | 3.5 | 1.7 | 5.7 |
| Females | | | | |
| Control | 3.8 | 3.7 | 2.2 | 5.5 |
| CO_2_ | 13.0 | 13.0 | 8.8 | 17.9 |
| BG-Lure | 2.6 | 2.5 | 1.4 | 3.9 |
| CO_2_ + BG-Lure | 6.4 | 6.3 | 2.6 | 12.3 |

Table S3. Coefficients of a multi hurdle-model averaging of apparent density for adult *Aedes aegypti* mosquitoes, fitted with data collected in 2020 (89 trapping sessions) during a field experiment in La Reunion. The hurdle model was made of (i) a sub-model of absence probability (logistic Bernoulli regression model, generating all the 0’s - i.e., the hurdle), and (ii) a sub-model of abundance (0-truncated negative binomial model, generating the strictly positive counts). The two sub-models were jointly fitted with a maximum likelihood method.

| Covariate | Estimated | Lower 95% limit | Upper 95% limit |
| --- | --- | --- | --- |
| Absence model | | | |
| co2yes:sexmale | 0.4965 | -0.6413 | 1.9614 |
| co2yes | 0.4733 | -0.4164 | 1.7636 |
| bglyes | 0.0470 | -0.2474 | 0.9700 |
| bglyes:co2yes | -0.0013 | -0.0131 | 0.0078 |
| bglyes:sexmale | -0.0018 | -0.0550 | 0.0528 |
| sexmale | -0.2379 | -1.0202 | 0.8061 |
| (Intercept) | -0.2609 | -1.2981 | 1.1406 |
| Density model | | | |
| (Intercept) | 1.6889 | 0.9410 | 2.2348 |
| bglyes:sexmale | 0.0165 | -0.0329 | 0.1052 |
| bglyes:co2yes | 0.0032 | -0.0132 | 0.0204 |
| co2yes | -0.0376 | -1.4346 | 1.2328 |
| bglyes | -0.0938 | -1.1569 | 0.1243 |
| sexmale | -0.3150 | -1.4742 | 1.0393 |
|  |  |  |  |
| co2yes:sexmale | -0.4409 | -1.6524 | 0.2059 |

Table S4. Coefficients of a multi hurdle-model averaging of apparent density for adult *Aedes albopictus* mosquitoes, fitted with data collected in 2020 (89 trapping sessions) during a field experiment in La Reunion. The hurdle model was made of (i) a sub-model of absence probability (logistic Bernoulli regression model, generating all the 0’s - i.e., the hurdle), and (ii) a sub-model of abundance (0-truncated negative binomial model, generating the strictly positive counts). The two sub-models were jointly fitted with a maximum likelihood method.

| Covariate | Estimated | Lower 95% limit | Upper 95% limit |
| --- | --- | --- | --- |
| Absence model | | | |
| co2yes:sexmale | 0.4965 | -0.6413 | 1.9614 |
| co2yes | 0.4733 | -0.4164 | 1.7636 |
| Bglyes | 0.0470 | -0.2474 | 0.9700 |
| bglyes:co2yes | -0.0013 | -0.0131 | 0.0078 |
| bglyes:sexmale | -0.0018 | -0.0550 | 0.0528 |
| Sexmale | -0.2379 | -1.0202 | 0.8061 |
| (Intercept) | -0.2609 | -1.2981 | 1.1406 |
| Density model | | | |
| (Intercept) | 1.6889 | 0.9410 | 2.2348 |
| bglyes:sexmale | 0.0165 | -0.0329 | 0.1052 |
| bglyes:co2yes | 0.0032 | -0.0132 | 0.0204 |
| co2yes | -0.0376 | -1.4346 | 1.2328 |
| Bglyes | -0.0938 | -1.1569 | 0.1243 |
| Sexmale | -0.3150 | -1.4742 | 1.0393 |
| co2yes:sexmale | -0.4409 | -1.6524 | 0.2059 |

| Table S5. Coefficients of a multi hurdle-model averaging of apparent density for adult *Culex qunquefasciatus* mosquitoes, fitted with data collected in 2020 (89 trapping sessions) during a field experiment in La Reunion. The hurdle model was made of (i) a sub-model of absence probability (logistic Bernoulli regression model, generating all the 0’s - i.e., the hurdle), and (ii) a sub-model of abundance (0-truncated negative binomial model, generating the strictly positive counts). The two sub-models were jointly fitted with a maximum likelihood method. | | | |
| --- | --- | --- | --- |
| Covariate | estimated | lower limit | upper limit |
| Absence model | | | |
| co2yes:sexmale | 0.4965 | -0.6413 | 1.9614 |
| co2yes | 0.4733 | -0.4164 | 1.7636 |
| Bglyes | 0.0470 | -0.2474 | 0.9700 |
| bglyes:co2yes | -0.0013 | -0.0131 | 0.0078 |
| bglyes:sexmale | -0.0018 | -0.0550 | 0.0528 |
| Sexmale | -0.2379 | -1.0202 | 0.8061 |
| (Intercept) | -0.2609 | -1.2981 | 1.1406 |
| Density model | | | |
| (Intercept) | 1.6889 | 0.9410 | 2.2348 |
| bglyes:sexmale | 0.0165 | -0.0329 | 0.1052 |
| bglyes:co2yes | 0.0032 | -0.0132 | 0.0204 |
| co2yes | -0.0376 | -1.4346 | 1.2328 |
| Bglyes | -0.0938 | -1.1569 | 0.1243 |
| Sexmale | -0.3150 | -1.4742 | 1.0393 |
| co2yes:sexmale | -0.4409 | -1.6524 | 0.205 |

# 3 Instructions to reproduce the analysis

The dataset, as well as the R code to reproduce the results presented in this paper, are available in a zipped file called bgtrap.zip. To reproduce the analysis (with a computer running MS Windows OS: not tested with other OS’s), use a computer with valid and updated installations of

- the R software, and at least the knitr and rmarkdown add-on packages, available at <https://cran.r-project.org/>
- Imagick and ImageMagick software: <https://github.com/Imagick/imagick>
- pandoc software: <https://pandoc.org/>
- unzip bgtrap.zip in an empty folder, making sure the sub-folder structure present in the zipped file is respected. You should get the following sub-folders and files:
  - in the root folder, (i) an rmarkdown file called bgtrap.Rmd containing the dataset as well as the master R code to produce the output, i.e. an HTML ioslides presentation called bgtrap.HTML, as well as graphic files, and HTML tables, (ii) an R file called make_bgtrap.R, and (iii) a configuration file _bookdown.yml.
  - a sub-folder called src, containing two R files: (i) a file packages.R: code to install, update, and load necessary add-on R packages, and (ii) a file functions.R: the R functions written for this analysis;
  - a sub-folder called data, containing a semi-colon separated text representation of the analysed dataset;
  - two empty sub-folders called tab and figs, to store the output.
- Either use R-Studio <https://www.rstudio.com/> to launch bgtrap.Rmd, or run make_bgtrap.R from within the folder where it lies. The analysis takes ca. 15 min. to complete with a DELL Laptop with co-processor Intel(R) Core(TM) i5-8350U CPU @ 1.70GHz 1.90 GHz, and 16 Go RAM.
- In case of difficulties to run the code, please contact the corresponding author, or the code author R. Lancelot ([renaud.lancelot@cirad.fr](mailto:renaud.lancelot@cirad.fr))
